# Improved Light Traps for Early Detection of Insect Pests of Phytosanitary Concern in Shipping Containers

**DOI:** 10.1093/jee/toab150

**Published:** 2021-07-29

**Authors:** Matteo Marchioro, Massimo Faccoli

**Affiliations:** Department of Agronomy, Food, Natural Resources, Animals and Environment (DAFNAE), University of Padua, Viale dell’Università, 16–35020 Legnaro (PD), Italy and

**Keywords:** alien species, interception, Coleoptera, Lepidoptera, Diptera

## Abstract

The number of introductions of alien insect has been increasing in the last decades, primarily transported in shipping containers. The attraction of light of different wavelengths (white, infrared, ultraviolet, and red) applied on sticky traps was tested for the development of new traps for hitchhiker insects. The addition of entomological glue and insecticide on the trap was also tested. Tests were conducted on *Cadra cautella* Walker (Lepidoptera: Pyralidae), *Drosophila melanogaster* Meigen (Diptera: Drosophilidae), *Sitophilus zeamais* Motschulsky (Coleoptera: Curculionidae), and *Tribolium castaneum* (Herbst) (Coleoptera: Tenebrionidae) and released inside a shipping container. In the first test, one light color at a time was tested setting eight traps in the container, one for each possible combination of the variables: light on or off, glue added or not, and insecticide sprayed or not. In the second, five traps were used, all of them coated with the entomological glue: one for each light color and one with light off as control. In all the single color tests (except for infrared), light-on traps captured more, except for *T. castaneum* that was not attracted to white. In the multi-color test, *C. cautella* showed no preference among white, ultraviolet, or red; *Drosophila melanogaster* preferred ultraviolet and white over red; and beetles had a much greater attraction to red. Lastly, the stronger entomological glue improved catches of beetles, whereas insecticides did not. In conclusion, results suggest a possible application of sticky light traps against hitchhiker insects and further studies should verify if the simultaneous use of different light colors can improve the trap performance and does not act as a repellent.

Introduction of non-native pests into new territories is a problem that has become of primary importance: driven by trade globalization, the rate of new introductions is increasing year by year (Bertelsmeier *et al.* 2017, Seebens *et al.* 2017). In the last centuries, human action has decisively facilitated and increased the processes of settlement of alien species outside their natural range (Hulme *et al.* 2008, Liebhold and Tobin 2008), with arthropods, and especially insects, considered as the most common and damaging group of invaders (Bradshaw *et al.* 2016). Invasion science is increasingly recognizing human-mediated dispersal as a pivotal node (Ricciardi *et al.* 2017, Bullock *et al.* 2018), demonstrating that the number of new biological invasions is closely related to the increase in international trade (Levine and D’Antonio 2003, Westphal *et al.* 2008). The most widely used means in international trade are shipping containers, which account for about 90% of global trade (IMO 2012, Bernhofen *et al.* 2016).

To try preventing and reducing new introductions, several international agreements have been signed such as the World Trade Organization Agreement on the Application of Sanitary and Phytosanitary Measures (SPS), the International Plant Protection Convention (IPPC) of the Food and Agricultural Organization of the United Nations, and the Convention for Biological Diversity (CBD). All these agreements are based on the assumption that prevention is the most economically sound way to manage biological invasions (Puth and Post 2005, Bogich *et al.* 2008, Hulme *et al.* 2009). Nevertheless, there are many major gaps in the regulatory framework for the management of invasive insects, mainly dealing with the difficulty in assessing the effect of potential preventive measures implemented to reduce the risk of new introductions (Hulme *et al.* 2008, Hulme 2009). In addition, due to the huge volumes of goods passing through points-of-entry every day, phytosanitary inspectors can only check a small part of the commodities, with increasing difficulties in selecting the loads to be sampled (Everett 2000, NRC 2002, Surkov *et al.* 2008).

The work of phytosanitary inspectors is a part of the *border surveillance*, applied at the point-of-entry, in order to prevent the settlement of alien species at the initial stage of their possible invasion process (Hulme 2014). In recent years, many tools and techniques have been tested to increase the effectiveness and efficiency of visual inspections (Augustin *et al.* 2012, Poland and Rassati 2019). Traps activated with pheromones, or volatiles, or other lures (e.g., light and colors) are the most common tools used in bio-surveillance programs, besides sniffer dogs, electronic noses, genetic tools for barcoding, acoustic detection, and laser vibrometry (Augustin *et al.* 2012, Poland and Rassati 2019). However, baited traps have a limit linked to the specificity of the pheromones used, which are often active only against one or a few species (Augustin *et al.* 2012, Rassati et al. 2015, 2019). Moreover, pheromone traps are active only during the flight dispersal of the insects in the new area, when adults have already left infested goods and containers. Therefore, traps baited using generic visual (Olenici *et al.* 2001, Sakalian and Mario 2004) or luminous stimuli (Ndengué *et al.* 2019, Silva *et al.* 2019) may have very high potentials in the early detection of unknown alien insect species arriving in international points-of-entry, especially when used inside the containers, i.e., before insect dispersal (Marchioro *et al.* 2020).

In the field of luminous stimuli, insects can be attracted (positive phototaxis) or repelled (negative phototaxis) to special light sources (Park and Lee 2017). Although the use of light is already widespread in integrated pest management (Garstang 2004), there is still no large scale application of light traps for the interception of alien species. In general, the vision of insect pests ranges from a wavelength of 350 nm (ultraviolet) to 700 nm (red; Land 1997). In light traps, incandescent or mercury vapor light bulbs are widely used, but LEDs (light emitting diodes) have been used increasingly in recent times (Oh 2011, Mangan and Chapa 2013, Park and Lee 2016). The advantages of LEDs are numerous and include small size, low weight, low electricity consumption, long lifetime, low temperature, high luminous efficiency, selectivity of specific wavelength, and light intensity (Cohnstaedt *et al.* 2008, Yeh and Chung 2009).

Widely used in agricultural systems (Oh 2011, Park and Lee 2016), light traps were also tested in border surveillance for the interception of pests transported with goods inside containers (Mangan and Chapa 2013, Marchioro *et al.* 2020). A research conducted by Marchioro *et al.* (2020) tested a light trap model inside a container, under different loading conditions, on four model species: *Cadra cautella* Walker (Lepidoptera: Pyralidae), *Drosophila melanogaster* Meigen (Diptera: Drosophilidae), *Sitophilus zeamais* Motschulsky, and *Ips typographus* L. (Coleoptera: Curculionidae). Results showed that trap performance is not affected by the container load and a high number of catches were recorded for Diptera and Lepidoptera. Instead, the trap was scarcely effective against beetles as the glue of the sticky cards of the trap was not strong enough to catch these insects, but a low attractiveness of the light installed in the trap also cannot be excluded. Results of this research have been encouraging and positive, but have also highlighted some gaps to be filled and improvements to be made on traps to improve their performance and effectiveness against more species. In view of these first results, the aim of this study was to investigate 1) how model species belonging to different insect orders respond to different light colors (i.e., wavelength), and 2) whether the synergistic use of more powerful glue and contact insecticides would improve capture performance of traps compared to the use of sticky cards only. This aims to develop a generic light trap efficient in early detection of alien insects belonging to different orders and families.

## Materials and Methods

### Tested Traps

**Fig. 1.**
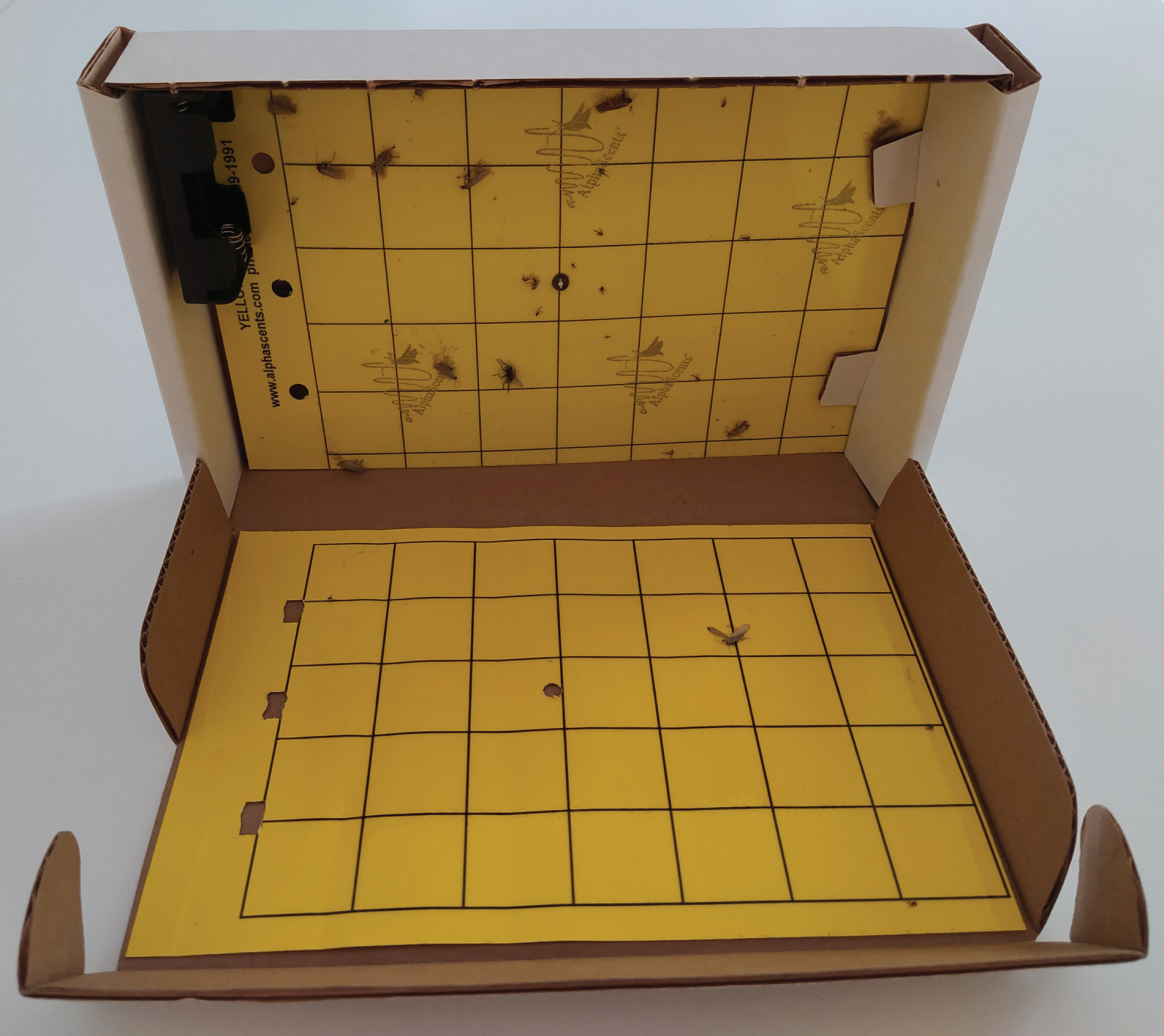
TransTrap, the trap used for the research.

Light-sticky traps (TransTrap, Alpha Scents Inc., West Linn, OR) developed for use inside containers during shipment were modified as shown by Marchioro *et al.* (2020). The original device consists of a small carton box (15 × 23 × 4 cm) containing a LED to attract insects and a yellow sticky card to catch them. The LED is powered by two AA batteries that can keep the light on for at least two consecutive weeks. The sticky card is attached to the bottom of the box and the light is positioned in the center. To increase the sticky surface and, consequently, the catching performance of the trap, we attached a second sticky card inside the box lid (Fig. 1). Sticky cards are produced by Alpha Scents Inc. too, and they are a standard model mainly indicated against flies, aphids, hoppers, psyllids, and yellow jackets (Alpha Scents Inc. 2013) and, also considering results obtained by Marchioro *et al.* (2020), they probably are not stronger enough in order to capture beetles.

A standard LED emits light that has two peaks, one at 465 nm (indigo) and the second between 525 and 600 nm (between green and yellow) and the result is white light. Beside the original trap model, in this study, we also replaced the manufacturer’s LED with LEDs of other three wavelengths: ultraviolet (wavelength 410 nm), red (wavelength 625 nm), and infrared (wavelength 940 nm). In order to prevent beetles from escaping, the inside surfaces of traps were also sprinkled with a strong entomological glue (Temo-O-Cid, Adama Italia s.r.l., Bergamo, Italy) and a solution composed by 1 ml of deltamethrin-based insecticide (Decis 15 EW, Bayer AG, Leverkusen, Germany) per 1 liter of water. Temo-O-Cid is a specific glue for the capture of flies and insects that can be spread with a brush. Once applied, the evaporation of the solvent contained makes the product absolutely nontoxic. It does not dry and retains its characteristics even when exposed to atmospheric agents. Temo-O-Cid is used to prepare chromotropic and all kinds of traps, to catch insects in orchards, vineyards, and flower crops. The greater strength of this glue, combined with a greater thickness of glue on the sticky card after its addition, should make it easier to catch larger insects.

### Model Species

The different trap models were tested against four model species belonging to Coleoptera, Lepidoptera, and Diptera orders, the three most common orders found inside shipping containers (Meurisse *et al.* 2019). *Sitophilus zeamais*, the maize weevil, is one of the major pests of stored maize in tropical and temperate regions of the world, but it also infests other cereals as alternative hosts (Erenso and Berhe 2016, Nwosu 2018). *Tribolium castaneum* (Herbst) (Coleoptera: Tenebrionidae), the red flour beetle, is a stored grain, flour, and other cereal product pest (Brown *et al.* 2009). *Cadra cautella*, the almond moth, is a pest of cereal grains, beans, and other dried seeds (Aldawood *et al.* 2013, Husain *et al.* 2017). *Drosophila melanogaster* is a fruit and vegetable pest (Mallis 1954, Birmingham *et al.* 2011).

All insects were provided by a laboratory (Entostudio s.r.l., Padua, Italy) specialized in the breeding of arthropod species for scientific purposes. The colony of *S. zeamais* was established in 2014 with insects collected in the field. Adults were bred in plastic cups enclosed by a net and fed with grain. The photoperiod lasted 12 h at a solar spectrum artificial light of 6,000 K and 300 lux intensity and they were bred at 25 ± 1°C and 50 ± 5% RH. Similarly, adults of *T. castaneum* were bred in plastic cups enclosed by a fine net, at 25 ± 1°C and 50 ± 5% RH. The photoperiod, at a solar spectrum artificial light of 6000 K and 300 lux intensity, lasted 14 h. Insects were fed with 95% of flour and 5% of beer yeast and a vial filled with water was present in the plastic cup to provide water and humidity to the colony. Adults of *C. cautella* were bred in glass jars positioned upside down with the opening closed by a 2-mm mesh net. The jar was placed above a plastic container to collect the eggs, which were then moved daily into plastic cups containing a mixture of wheat and corn flour, oat, bran, dry fruit, glycerol, honey, and yeast. The insects were reared at 25 ± 1°C and 50 ± 5% RH. The photoperiod, at a solar spectrum artificial light of 6,000 and 300 lux intensity, lasted 12 h. Adults of *D. melanogaster* were bred in BugDorme cages. A mixture of water, pieces of potato and fruit, powdered milk, and sugar was used as food and as an oviposition substrate. Insects were reared at 25 ± 1°C and 50 ± 5% of RH with a photoperiod of 12 h at a solar spectrum artificial light of 6,000 K and 300 lux intensity.

All insects were tested only once and within 2 d after their emergence (for *C. cautella* and *D. melanogaster*) to guarantee highest vitality. We assumed a sex-ratio 1:1 as these four species reproduce sexually and produce a sex-balanced offspring (Englert and Bell 1962, Santos *et al.* 1994, Danho *et al.* 2002, Soffan *et al.* 2012). The insects used in each trial were chosen randomly.

### Trials in Container

Trials were conducted in an ISO standard shipping container 1CC (interior size: 5.8 m length, 2.3 m width, and 2.3 m height; ISO 2013). The container was placed in the Agripolis Campus, University of Padua (Legnaro, Italy), without any shelter from sun and rain. The container was empty of goods and only the traps and insect-releasing device were placed inside. In contrast to the tests conducted the year before (Marchioro *et al.* 2020), in this case, no container load tests were carried out, as the aim of the study was to test the attractiveness of different wavelengths. Traps were positioned inside the container open, with lid and box forming a 90° angle. The lid was resting on the ground while the box was in a vertical position, as can be seen in Fig. 1. In trials in which some traps had to be placed on the top of the container, the use of metal hooks made it possible to maintain the same conformation as traps placed on the ground. Tests were conducted between May and July 2020.

#### Single Color Tests

**Fig. 2.**
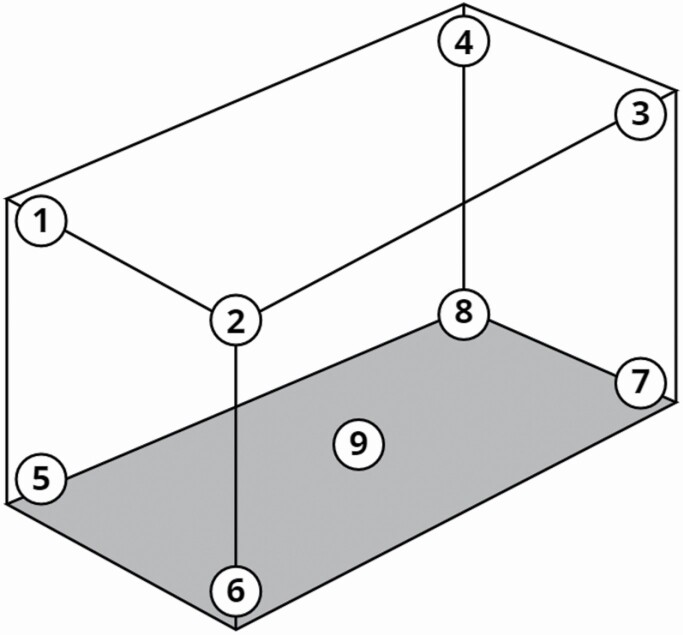
Disposition of the traps inside the container (doors were on the left side). Single color test: 1–8. Multicolor test: 5–9.

The first group of tests was conducted using only one light color at a time. We used eight different traps at the same time, one for each of the eight possible combinations of the three considered variables: light (turned on or off), additional glue (added or not), and insecticide (sprayed or not). A trap with a turned off light and without additional glue or insecticide was used as control (trap ‘C’). Each different combination of variables corresponds to a different code: ‘L’ if the trap light was on, ‘G’ if glue was added, and ‘I’ if insecticide was added. The eight traps were randomly set in the eight corners of the container (changing the traps arrangement at each trial; Fig. 2): four traps were laid on the ground while four were hung by hooks from the ceiling. During each trial, we used 50 individuals for each model species released at the same time, for a total of 200 insects. With a device consisting of a cup containing the insects and a rope tied to the lid to free them, it was possible to release the insects just before the doors of the container were closed to prevent their escape. For each LED color (white, infrared, ultraviolet, and red), we conducted seven repetitions, on seven consecutive nights with similar weather conditions; each repetition lasted 18 h (from 05:00 p.m. to 11:00 a.m. the following day). At the end of each trial, before starting a new one, we ventilated the container, swept the floor, and removed all insects from the walls to make sure there were none left inside.

#### Multicolor Tests

Other tests were conducted using, at the same time in the container, all traps with the four different light colors. One trap per light color (white, red, ultraviolet, and infrared) and coated with entomological glue was tested, whereas a trap with a turned off light and without additional glue was used as control (trap ‘C’). Again, each different light color corresponds to a different code: ‘W’ for white light, ‘IR’ for infrared light, ‘UV’ for ultraviolet light, and ‘R’ for red light. The five traps were randomly set inside a container, on the floor (changing the traps arrangement at each trial; Fig. 2). Seven repetitions were conducted on seven consecutive nights, with duration of 18 h (from 05:00 p.m. to 11:00 a.m. the following day). Fifty individuals per model species were used in each repetition, for a total of 200 insects per day.

### Statistical Analysis

In the ‘single color tests’, mean catches per trap of the model species were compared using a mixed-effect model, with trap type (the eight possible combinations of the three tested variables) as a fixed variable and repetitions as a random variable. The model was fitted using the ‘lmer’ or ‘glmer’ functions in the lme4 package (Bates *et al.* 2015) and using Poisson distribution or logarithmic transformation as appropriate (Table 1). Multiple comparisons between fixed variables were obtained using Tukey’s test (‘emmeans’ function in the emmeans package) with ‘Bonferroni correction’ (Russell 2019). When the use of this statistical test was inapplicable because of few captures, the Kruskal–Wallis test was applied using the ‘kruskal.test’ function in the stat package (R Core Team 2019).

**Table 1.** Results of the statistical models (*P*-value) used to test the effect of trap type for the four model species in all the tests conducted

Model species	Trap type	*P*-value	t/z-value	df	Model	Distribution
Single color test—White light						
*Cadra cautella*	L	<0.001	9.303	48	LMM	Normal
	L + G	<0.001	7.048			
	L + I	<0.001	7.330			
	L + G + I	<0.001	7.893			
*Drosophila melanogaster*	L	<0.001	4.878	47	GLMM	Poisson
	L + G	<0.001	3.826			
	L + I	<0.001	4.334			
	L + G + I	<0.001	4.366			
Single color test—Infrared light						
*Cadra cautella*	–	–	–	47	GLMM	Poisson
*Drosophila melanogaster*	–	–	–	47	GLMM	Poisson
Single color test—Ultraviolet light						
*Cadra cautella*	L	<0.001	3.660	48	LMM	Normal
	L + G	<0.01	3.253			
	L + I	<0.01	3.186			
	L + G + I	<0.01	3.253			
*Drosophila melanogaster*	L	<0.001	4.369	47	GLMM	Poisson
	L + G	<0.001	4.872			
	L + I	<0.001	5.546			
	L + G + I	<0.001	6.097			
Single color test—Red light						
*Cadra cautella*	L	<0.01	3.254	47	GLMM	Poisson
	L + G	<0.01	3.254			
	L + I	<0.001	3.531			
	L + G + I	<0.001	4.542			
*Drosophila melanogaster*	L	<0.001	4.777	48	LMM	Log-transf.
	L + G	<0.001	4.245			
	L + I	<0.001	5.552			
	L + G + I	<0.001	5.098			
Multi-color test						
*Cadra cautella*	UV	<0.001	3.791	24	LMM	Normal
*Drosophila melanogaster*	W	<0.001	6.732	29	GLMM	Poisson
	UV	<0.001	5.988			
	R	<0.001	4.159			
*Sitophilus zeamais*	W	<0.001	5.727	24	LMM	Log-transf.
	UV	<0.01	3.279			
	R	<0.001	10.370			
*Tribolium castaneum*	W	<0.05	2.621	24	LMM	Log-transf.
	UV	<0.001	7.474			
	R	<0.001	13.131			

L = light on; G = glue added; I = insecticide sprayed.

Models = LMM: linear mixed-effects model; GLMM: generalized linear mixed-effects model; Distribution = Normal: normal distribution; Log-transf.: normal on log-transformed data; Poisson: Poisson distribution. *t*-value is referred to LMM models; *z*-value is referred to GLMM models.

In the ‘Multi color tests’, mean catches per trap of the model species were compared using a mixed-effect model, with trap type (the five light colors, including control) as a fixed variable and repetitions as a random variable; Tukey’s test with ‘Bonferroni correction’ was used for multiple comparisons between fixed variables. Statistical analysis was performed using R software, version 3.6.1 (R Core Team, 2019).

## Results

The main obtained results are presented here briefly according to the tested light color. The number of captures for each model species in each test is reported in Supp Table S1 (online only).

### Single Color Test—White Light

**Fig. 3.**
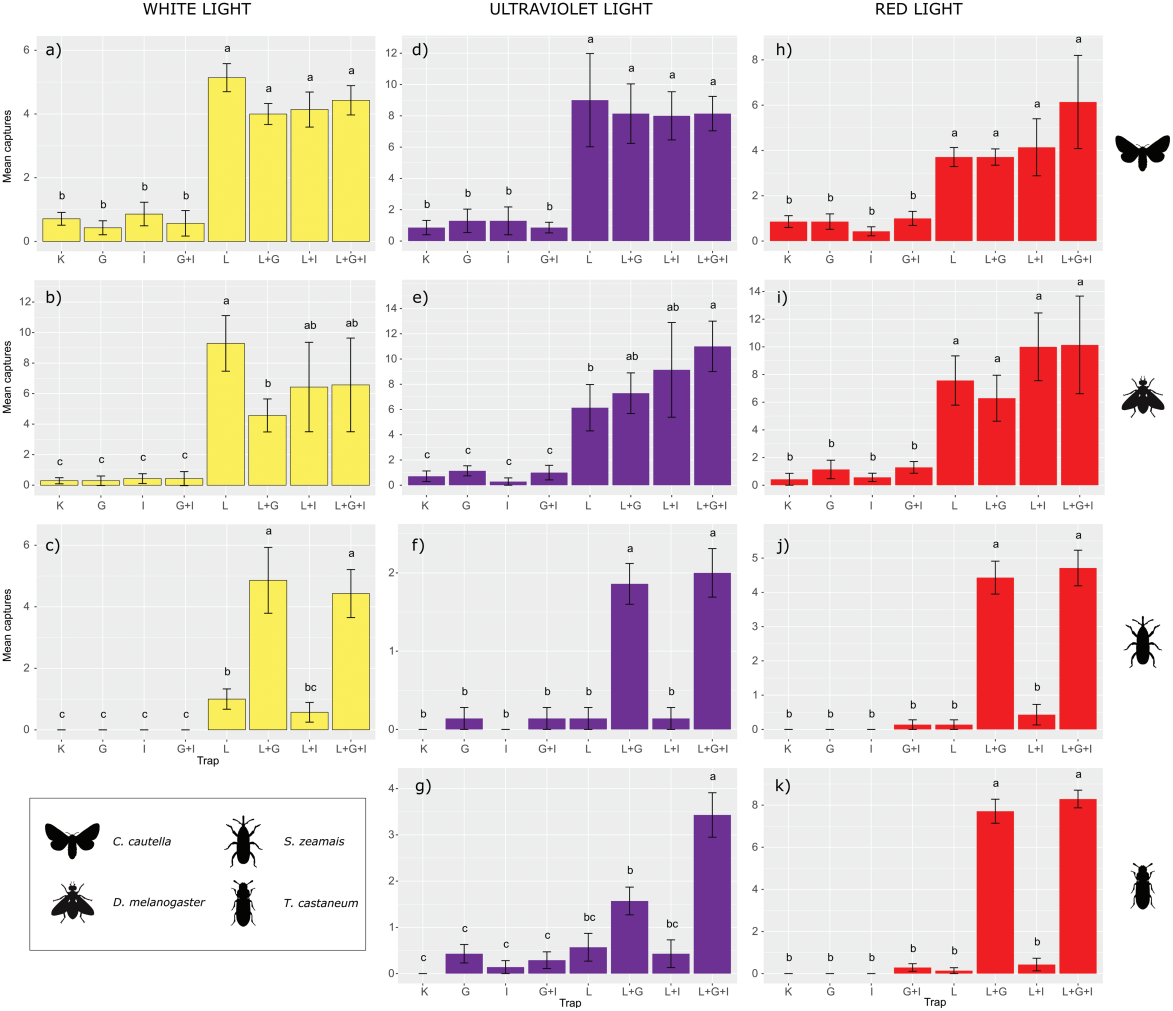
Mean (± SE) number of insects captured by each trap combination during the ‘Single color tests’ and divided for each model species (rows) and light color (columns). Means with different letters on the same graph were significantly different.

The four trap combinations with light turned on (trap L, L + G, L + I, and L + G + I) caught similar numbers of *C. cautella* and significantly higher than the light-off traps (C, G, I, and G + I; Table 1; Supp Table S2 [online only]; Fig. 3a). The same result was observed for *D. melanogaster*, although in this species trap L captured significantly more individuals than L + G (Table 1; Supp Table S2 [online only]; Fig. 3b). *Sitophilus zeamais* was captured significantly more in traps L + G and L + G + I than all the others. Moreover, in *S. zeamais* the other two light-on traps (L and L + I) caught significantly more than light-off traps (Supp Table S3 [online only]; Fig. 3c). Only one individual of *T. castaneum* was captured in traps L and L + G + I, and numbers too low to allow statistical analysis.

### Single Color Test—Infrared Light

The four model species were captured only in very low numbers in traps activated with infrared light. Although for beetles (*S. zeamais* and *T. castaneum*) there were a few captures in traps treated with additional glue or insecticide (G, G + I, L + G, L + I, and L + G + I), there were no significant differences between the eight tested trap models (Supp Tables S2 and S3 [online only]).

### Single Color Test—Ultraviolet Light

Similar to the white light test, *C. cautella* and *D. melanogaster* were caught significantly more by light-on traps (L, L + G, L + I, and L + G + I) than light-off traps (Table 1; Supp Table S2 [online only]; Fig. 3d). In addition, for *D. melanogaster*, L + G + I captured significantly more individuals than L traps (Table 1; Supp Table S2 [online only]; Fig. 3e). With beetles (*S. zeamais* and *T. castaneum*), L + G and L + G + I captured significantly more insects than the other trap models (Table 1). Although for *S. zeamais* the other six trap models showed no significant differences (with C and I trapping no individuals [Supp Table S3 [online only]; Fig. 3f]), for *T. castaneum* L + G + I was the trap type that captured the largest number of insects, whereas L + G captured significantly more than C, G, I, and G + I traps (Supp Table S3 [online only]; Fig. 3g).

### Single Color Test—Red Light

Again, *C. cautella* and *D. melanogaster* were captured significantly more by light-on (L, L + G, L + I, and L + G + I) than light-off traps (Table 1; Supp Table S2 [online only]; Fig. 3h–i). Similarly, light-on traps coated with additional glue (L + G and L + G + I) caught significantly more individuals of *S. zeamais* (*P* < 0.001, *K* = 46.290) and *T. castaneum* (*P* < 0.001, *K* = 45.231); in both beetle species, C, G, and I traps captured no insects (Supp Table S3 [online only]; Fig. 3j–k).

### Multicolor Tests

**Fig. 4.**
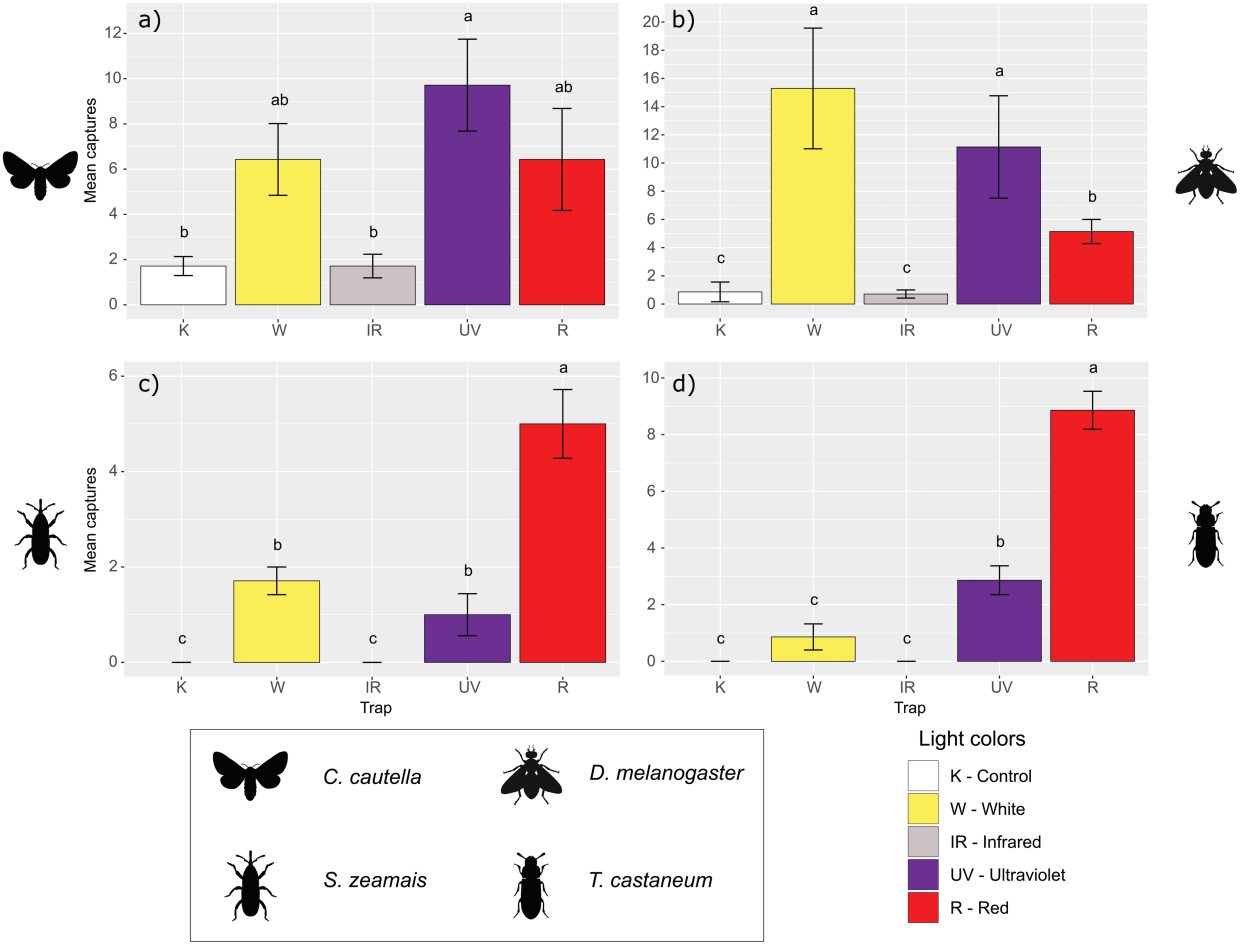
Mean (± SE) number of insects, divided for each model species, captured by each light color during the ‘Multi-color test’. Means with different letters on the same graph were significantly different.

Captures of *C. cautella* in ultraviolet light trap were significantly higher than in control (light-off trap) and infrared light traps, but without differences from white and red light traps (Table 1; Supp Table S2 [online only]; Fig. 4a). For *D. melanogaster* white, ultraviolet and red light traps caught a significantly higher number of individuals than control and infrared light traps. Moreover, white and ultraviolet light traps captured more than the red one (Table 1; Supp Table S2 [online only]; Fig. 4b). Lastly, for *S. zeamais* and *T. castaneum*, red light trap outperformed the others. Ultraviolet and white light traps caught significantly more individuals of *S. zeamais* than control and infrared light traps (Table 1; Supp Table S2 [online only]; Fig. 4c), whereas for *T. castaneum*, ultraviolet light trap outperformed control, infrared, and white traps (Table 1; Supp Table S2 [online only]; Fig. 4d).

## Discussion

Results show different phototactic responses for the various tested species. All model species showed a general attraction to light: in fact, in all the single color tests (except for infrared light) light-on traps captured more specimens. Only *T. castaneum* did not present an attraction for white light. In particular, in the multi-color test, we found that *C. cautella* has no preference between white, ultraviolet, and red lights; *Drosophila melanogaster* prefers ultraviolet and white over red light; *Sitophilus zeamais* and *T. castaneum* have a much greater attraction to red light.

Land (1997) observed that, in general, insects can perceive light ranging in wavelength from 350 (ultraviolet) to 700 nm (red) and results of the ‘single-color tests’ agree with him. In fact, for all four model species, we obtained a significant effect of light with ultraviolet, white, and red wavelengths, but not with infrared (940 nm). Moreover, light-on traps (with white, ultraviolet, and red LED) with the addition of entomological glue captured significantly more beetles (both *S. zeamais* and *T. castaneum*) than normal traps or traps with insecticide only. This result confirms the hypothesis formulated by Marchioro *et al.* (2020) according to which the standard glue of sticky cards, alone, was unsuitable to retain trapped beetles. Adding insecticide does not improve trap performance, probably because beetles are able to escape before dying. This is also true for Lepidoptera and Diptera: in fact, captures of light-on traps with insecticide are similar to other light-on traps. However, avoiding the use of insecticides may also allow trap use in containers transporting food, without risk of goods contamination.

White light shows among the best results for catching Lepidoptera and Diptera (although with no significant differences from ultraviolet and red light), probably due to its composition of two peaks at indigo and green-yellow wavelength. Measures of spectral efficiency of *C. cautella*, in fact, highlight two regions of high efficiency at 546 nm (yellow-green) and 350 nm (ultraviolet) (Gilburt and Anderson 1996). Moreover, it has been observed by numerous studies that green and blue lights are very effective in catching many Lepidoptera, like for instance *Ephestia kuehniella* (Soderstrom 1970), *Plodia interpunctella* (Soderstrom 1970, Park and Lee 2016), *Sitotroga cerearella* (Soderstrom 1970), *Spodoptera exigua* (Oh 2011), *Spodoptera litura* (Yang *et al.* 2012), and *Plutella xylostella* (Cho and Lee 2012). Also *D. melanogaster* is most sensitive to short wavelength lights (ultraviolet, blue, and green) with two peaks at 420 nm and 495 nm (de Salomon and Spatz 1983, Kelber and Henze 2013). Light traps with similar wavelengths are largely used for moth monitoring, but they may also intercept Diptera (Kim and Lee 2014a, da Silva *et al.* 2019, Ndengué *et al.* 2019, Silva *et al.* 2019).

Ultraviolet was one of the best wavelengths for Lepidoptera and Diptera (although catches did not differ from those obtained with white and red light), while it provided scarce results for Coleoptera. The general effectiveness of UV as an attraction for several insects is well-known (Hollingsworth *et al.* 1968, Kirkpatrick *et al.* 1970, van Grunsven *et al.* 2014, Thein and Choi 2016), in particular, for moths (Cho and Lee 2012, Infusino *et al.* 2017) and flies (Gaglio *et al.* 2018, Hogsette 2019). Regarding *S. zeamais* and *T. castaneum*, the literature instead provides conflicting results about the attractiveness of UV. On one hand, Duehl *et al.* (2011) found that *T. castaneum* was most attracted by UV wavelength and Kirkpatrick *et al.* (1970) found that some species of stored-products beetles preferred UV over green light. On the other hand, Park *et al.* (2015) and Song *et al.* (2016a) found that UV light was the less attractive for *S. zeamais* and *T. castaneum*.

Red wavelength also showed high attractiveness for our model species. For *C. cautella*, the number of trapped insects was similar to white and ultraviolet lights, as found for the similar species *P. interpunctella* (Park and Lee 2016). However, for other moth species belonging to different orders, results are different: red light is less attractive than other light colors in *S. litura* (trapped with blue and green [Yang *et al.* 2012]), *S. exigua* (trapped with white light [Oh 2011]), and *S. cerearella* (trapped with ultraviolet light [Kim and Lee 2014b]). For *D. melanogaster* captures obtained with red light were lower than white and ultraviolet lights. Also for another Dipteran, *Liriomyza trifolii*, red light was less attractive than green and yellow lights, but more attractive than ultraviolet (Kim and Lee 2014a). Finally, for both beetle species, red light was the most attractive one, with more than twice the catches than those of white and ultraviolet. These results agree with other researches conducted on the phototactic behaviour of *S. zeamais* (Park *et al.* 2015) and *T. castaneum* (Song et al. 2016a, 2016b), where red light was the best wavelength for both species. However, different results were obtained for other beetles: *S. oryzae*, congeneric of *S. zeamais*, preferred blue and green lights, whereas red and ultraviolet lights showed similarly lower capture performance (Jeon *et al.* 2012).

Finally, in our trials, infrared light was not attractive to any of the tested species. This result is not surprising as insect vision is generally shifted towards ultraviolet and they seem unable to see infrared radiation (Land, 1997). Other studies dealing with the phototactic behaviour of fly and moth species confirm this observation (Cho and Lee 2012, Kim and Lee 2014a, 2014b, Park and Lee 2016). However, certain studies have shown a similar attraction of *S. zeamais* to red, yellow, and infrared light (Park *et al.* 2015), and of *T. castaneum* similar to infrared, white, yellow, green, and blue lights, and higher than ultraviolet (Song *et al.* 2016a).

In conclusion, we found that light is an effective ‘broad-spectrum’ attractant for several insect species belonging to different orders. Moreover, the use of a stronger glue on the sticky cards improves captures of beetles (although it does not improve moth and fly catches), solving the problem highlighted by Marchioro *et al.* (2020). Instead, the insecticide, in the formulations and doses tested, does not give any improvement in terms of catches. However, we also found that there is a clear response of the different species to the different lights tested: white and ultraviolet lights are the most attractive for *C. cautella* and *D. melanogaster*, while red is the most effective in catching beetles. Moreover, we can hypothesize that using at the same time different traps with different light colors, there must have been some interference in the case of two colors both attractive to one species. Probably, using only one trap, the trap performance will increase. A possible solution could consist in the use of different lights at the same time in the same trap, but further studies should verify that this combination can improve the trap performance and is not a repellent. The aim of this study is to find a trap that can be used in a wide range of shipments, with a wide variety of commodities. The tested glue (Temo-O-Cid) is non-toxic and this allows the trap to be used in conjunction with any type of food product (grains, flours, fruits, and vegetables). However, it can be used with any kind of cargo that can carry hitchhikers’ insects. This is only a pilot study that used few model species. In order to obtain more comprehensive and reliable results, other tests must be conducted, possibly during real shipments.

## Supplementary Material

toab150_suppl_Supplementary_materials
