# Supplementary material for: Improved Light Traps for Early Detection of Insect Pests of Phytosanitary Concern in Shipping Containers
Source: J Econ Entomol. 2021 Jul 29;114(5):2060–8. doi: 10.1093/jee/toab150 (PMC8513576; doi:10.1093/jee/toab150)
Supplement: toab150_suppl_Supplementary_materials [file toab150_suppl_supplementary_materials.docx]

**SUPPLEMENTARY**

**Table S1.** Mean (± SE) number of insects captured by each trap combination during the “Single color tests”, divided for each light color. C = control; L = light on; G = glue added; I = insecticide sprayed

|  |  | C | G | I | L | L + G | L + I | G + I | L + G + I |
| --- | --- | --- | --- | --- | --- | --- | --- | --- | --- |
| WHITE | *Cadra cautella* Walker | 0.71 ± 0.18 | 0.42 ± 0.20 | 0.85 ± 0.34 | 5.14 ± 0.40 | 4 ± 0.30 | 4.14 ± 0.50 | 0.57 ± 0.36 | 4.42 ± 0.42 |
|  | *Drosophila melanogaster* Meigen | 0.28 ± 0.18 | 0.28 ± 0.28 | 0.42 ± 0.29 | 9.28 ± 1.68 | 4.57 ± 0.99 | 6.42 ± 2.71 | 0.42 ± 0.42 | 6.57 ± 2.84 |
|  | *Sitophilus zeamais* Motschulsky | - | - | - | 1.00 ± 0.30 | 4.85 ± 0.98 | 0.57 ± 0.29 | - | 4.42 ± 0.71 |
|  | *Triboliu castaneum* (Herbst) | - | - | - | 0.14 ± 0.14 | - | - | - | 0.14 ± 0.14 |
| INFRARED | *Cadra cautella* Walker | 1.42 ± 0.52 | 1.14 ± 0.55 | 1.85 ± 0.76 | 1.28 ± 0.60 | 1.14 ± 0.50 | 1.71 ± 0.42 | 1.71 ± 0.35 | 2.28 ± 0.60 |
|  | *Drosophila melanogaster* Meigen | 1.57 ± 0.71 | 2.42 ± 0.57 | 2.00 ± 0.48 | 1.42 ± 0.71 | 2.14 ± 0.55 | 1.42 ± 0.64 | 1.85 ± 0.70 | 2.28 ± 0.56 |
|  | *Sitophilus zeamais* Motschulsky | - | 0.28 ± 0.18 | - | - | - | - | 0.28 ± 0.28 | 0.28 ± 0.28 |
|  | *Triboliu castaneum* (Herbst) | - | 0.57 ± 0.29 | - | - | 0.14 ± 0.14 | 0.28 ± 0.28 | - | 1.00 ± 0.48 |
| ULTRAVIOLET | *Cadra cautella* Walker | 2.00 ± 1.52 | 1.28 ± 0.74 | 1.28 ± 0.89 | 9.00 ± 2.98 | 7.00 ± 2.26 | 6.71 ± 2.02 | 0.85 ± 0.34 | 6.00 ± 1.77 |
|  | *Drosophila melanogaster* Meigen | 0.71 ± 0.42 | 1.14 ± 0.40 | 0.28 ± 0.28 | 6.14 ± 1.84 | 7.28 ± 1.61 | 9.14 ± 3.75 | 1.00 ± 0.57 | 11.00 ± 2.00 |
|  | *Sitophilus zeamais* Motschulsky | - | 0.14 ± 0.14 | - | 0.14 ± 0.14 | 1.85 ± 0.26 | 0.14 ± 0.14 | 0.14 ± 0.14 | 2.00 ± 0.30 |
|  | *Triboliu castaneum* (Herbst) | - | 0.42 ± 0.20 | 0.14 ± 0.14 | 0.57 ± 0.29 | 1.57 ± 0.29 | 0.42 ± 0.29 | 0.28 ± 0.18 | 2.42 ± 0.71 |
| RED | *Cadra cautella* Walker | 0.85 ± 0.26 | 0.85 ± 0.34 | 0.42 ± 0.20 | 3.71 ± 0.42 | 3.28 ± 0.42 | 4.14 ± 1.26 | 1.00 ± 0.30 | 6.14 ± 2.06 |
|  | *Drosophila melanogaster* Meigen | 0.42 ± 0.42 | 1.14 ± 0.67 | 0.57 ± 0.29 | 5.57 ± 2.22 | 6.28 ± 1.65 | 8.14 ± 3.00 | 1.28 ± 0.42 | 10.10 ± 3.52 |
|  | *Sitophilus zeamais* Motschulsky | - | - | - | 0.14 ± 0.14 | 4.42 ± 0.48 | 0.42 ± 0.29 | 0.14 ± 0.14 | 4.71 ± 0.52 |
|  | *Triboliu castaneum* (Herbst) | - | - | - | 0.14 ± 0.14 | 7.71 ± 0.56 | 0.42 ± 0.29 | 0.28 ± 0.18 | 8.28 ± 0.42 |

**Table S2.** Output (P-value) of the multiple comparisons between different trap combinations conducted with Tukey’s test in the “Single color tests” and “Multi-color test”. * P < 0.05; ** P < 0.01; *** P < 0.001. Single color tests: C = control; L = light on; G = glue added; I = insecticide sprayed. Multi-color test: C = control; W = white light; IR = infrared light; UV = ultraviolet light; R = red light.

| Model species |  | C | G | | I | L | | L+G | L+I | | G+I |
| --- | --- | --- | --- | --- | --- | --- | --- | --- | --- | --- | --- |
| Single color test - White light | | | | | | | | | | | |
| *Cadra cautella* | G | - |  | |  |  | |  |  | |  |
|  | I | - | - | |  |  | |  |  | |  |
|  | L | *** | *** | | *** |  | |  |  | |  |
|  | L+G | *** | *** | | *** | 0.823 | |  |  | |  |
|  | L+I | *** | *** | | *** | - | | - |  | |  |
|  | G+I | - | - | | - | *** | | *** | *** | |  |
|  | L+G+I | *** | *** | | *** | - | | - | - | | *** |
| *Drosophila melanogaster* | G | - |  | |  |  | |  |  | |  |
|  | I | - | - | |  |  | |  |  | |  |
|  | L | *** | *** | | *** |  | |  |  | |  |
|  | L+G | ** | ** | | ** | * | |  |  | |  |
|  | L+I | *** | *** | | *** | - | | - |  | |  |
|  | G+I | - | - | | - | *** | | ** | *** | |  |
|  | L+G+I | *** | *** | | *** | - | | - | - | | *** |
| Single color test - Infrared light | | | | | | | | | | | |
| *Cadra cautella* | G | - |  | |  |  | |  |  | |  |
|  | I | - | - | |  |  | |  |  | |  |
|  | L | - | - | | - |  | |  |  | |  |
|  | L+G | - | - | | - | - | |  |  | |  |
|  | L+I | - | - | | - | - | | - |  | |  |
|  | G+I | - | - | | - | - | | - | - | |  |
|  | L+G+I | - | - | | - | - | | - | - | | - |
| *Drosophila melanogaster* | G | - |  | |  |  | |  |  | |  |
|  | I | - | - | |  |  | |  |  | |  |
|  | L | - | - | | - |  | |  |  | |  |
|  | L+G | - | - | | - | - | |  |  | |  |
|  | L+I | - | - | | - | - | | - |  | |  |
|  | G+I | - | - | | - | - | | - | - | |  |
|  | L+G+I | - | - | | - | - | | - | - | | - |
| Single color test - Ultraviolet light | | | | | | | | | | | |
| *Cadra cautella* | G | - |  | |  |  | |  |  | |  |
|  | I | - | - | |  |  | |  |  | |  |
|  | L | * | * | | * |  | |  |  | |  |
|  | L+G | * | * | | * | - | |  |  | |  |
|  | L+I | * | * | | * | - | | - |  | |  |
|  | G+I | - | - | | - | * | | * | * | |  |
|  | L+G+I | * | * | | * | - | | - | - | | * |
| *Drosophila melanogaster* | G | - |  | |  |  | |  |  | |  |
|  | I | - | - | |  |  | |  |  | |  |
|  | L | *** | *** | | *** |  | |  |  | |  |
|  | L+G | *** | *** | | *** | - | |  |  | |  |
|  | L+I | *** | *** | | *** | - | | - |  | |  |
|  | G+I | - | - | | - | *** | | *** | *** | |  |
|  | L+G+I | *** | *** | | *** | 0.062 | | 0.629 | - | | *** |
| Single color test - Red light | | | | | | | | | | | |
| *Cadra cautella* | G | - |  | |  |  | |  |  | |  |
|  | I | - | - | |  |  | |  |  | |  |
|  | L | * | * | | * |  | |  |  | |  |
|  | L+G | * | * | | * | - | |  |  | |  |
|  | L+I | * | * | | ** | - | | - |  | |  |
|  | G+I | - | - | | - | * | | * | * | |  |
|  | L+G+I | *** | *** | | *** | - | | - | - | | *** |
| *Drosophila melanogaster* | G | - |  | |  |  | |  |  | |  |
|  | I | - | - | |  |  | |  |  | |  |
|  | L | *** | *** | | *** |  | |  |  | |  |
|  | L+G | *** | ** | | *** | - | |  |  | |  |
|  | L+I | *** | *** | | *** | - | | - |  | |  |
|  | G+I | - | - | | - | ** | | * | *** | |  |
|  | L+G+I | *** | *** | | *** | - | | - | - | | ** |
| Multi-color test |  | C | | W | | | IR | | | UV | |
| *Cadra cautella* | W | 0.351 | |  | | |  | | |  | |
|  | IR | - | | 0.351 | | |  | | |  | |
|  | UV | ** | | - | | | ** | | |  | |
|  | R | 0.351 | | - | | | 0.351 | | | - | |
| *Drosophila melanogaster* | W | *** | |  | | |  | | |  | |
|  | IR | - | | *** | | |  | | |  | |
|  | UV | *** | | 0.328 | | | *** | | |  | |
|  | R | *** | | *** | | | *** | | | ** | |
| *Sitophilus zeamais* | W | *** | |  | | |  | | |  | |
|  | IR | - | | *** | | |  | | |  | |
|  | UV | * | | 0.220 | | | * | | |  | |
|  | R | *** | | ** | | | *** | | | *** | |
| *Tribolium castaneum* | W | 0.150 | |  | | |  | | |  | |
|  | IR | - | | 0.150 | | |  | | |  | |
|  | UV | *** | | *** | | | *** | | |  | |
|  | R | *** | | *** | | | *** | | | *** | |

**Table S3.** Output (P-value) of the multiple comparisons between different trap combinations conducted with Kruskal-Wallis test in the “Single color tests”. * P < 0.05; ** P < 0.01. C = control; L = light on; G = glue added; I = insecticide sprayed.

| Model species |  | C | G | I | L | L+G | L+I | G+I |
| --- | --- | --- | --- | --- | --- | --- | --- | --- |
| Single color test - White light | | | | | | | | |
| *Sitophilus zeamais* | G | - |  |  |  |  |  |  |
|  | I | - | - |  |  |  |  |  |
|  | L | * | * | * |  |  |  |  |
|  | L+G | ** | ** | ** | * |  |  |  |
|  | L+I | 0.08 | 0.08 | 0.82 | 0.35 | * |  |  |
|  | G+I | - | - | - | * | ** | 0.08 |  |
|  | L+G+I | ** | ** | ** | ** | 0.56 | ** | ** |
| Single color test - Infrared light | | | | | | | | |
| *Sitophilus zeamais* | G | 0.47 |  |  |  |  |  |  |
|  | I | - | 0.47 |  |  |  |  |  |
|  | L | - | 0.47 | - |  |  |  |  |
|  | L+G | - | 0.47 | - | - |  |  |  |
|  | L+I | - | 0.47 | - | - | - |  |  |
|  | G+I | 0.47 | 0.76 | 0.47 | 0.47 | 0.47 | 0.47 |  |
|  | L+G+I | 0.47 | 0.76 | 0.47 | 0.47 | 0.47 | 0.47 | - |
| *Tribolium castaneum* | G | 0.21 |  |  |  |  |  |  |
|  | I | - | 0.21 |  |  |  |  |  |
|  | L | - | 0.21 | - |  |  |  |  |
|  | L+G | 0.43 | 0.43 | 0.43 | 0.43 |  |  |  |
|  | L+I | 0.43 | 0.43 | 0.43 | 0.43 | - |  |  |
|  | G+I | - | 0.21 | - | - | 0.43 | 0.43 |  |
|  | L+G+I | 0.21 | 0.70 | 0.21 | 0.21 | 0.43 | 0.43 | 0.21 |
| Single color test - Ultraviolet light | | | | | | | | |
| *Sitophilus zeamais* | G | 0.53 |  |  |  |  |  |  |
|  | I | - | 0.53 |  |  |  |  |  |
|  | L | 0.53 | - | 0.53 |  |  |  |  |
|  | L+G | ** | ** | ** | ** |  |  |  |
|  | L+I | 0.53 | - | 0.53 | - | ** |  |  |
|  | G+I | 0.53 | - | 0.53 | - | ** | - |  |
|  | L+G+I | ** | ** | ** | ** | - | ** | ** |
| *Tribolium castaneum* | G | 0.14 |  |  |  |  |  |  |
|  | I | 0.55 | 0.43 |  |  |  |  |  |
|  | L | 0.14 | 0.92 | 0.40 |  |  |  |  |
|  | L+G | ** | * | ** | 0.09 |  |  |  |
|  | L+I | 0.29 | 0.88 | 0.69 | 0.79 | 0.05 |  |  |
|  | G+I | 0.29 | 0.75 | 0.72 | 0.69 | * | 0.94 |  |
|  | L+G+I | ** | ** | ** | ** | * | ** | ** |
| Single color test - Red light | | | | | | | | |
| *Sitophilus zeamais* | G | - |  |  |  |  |  |  |
|  | I | - | - |  |  |  |  |  |
|  | L | 0.47 | 0.47 | 0.47 |  |  |  |  |
|  | L+G | ** | ** | ** | ** |  |  |  |
|  | L+I | 0.29 | 0.29 | 0.29 | 0.58 | ** |  |  |
|  | G+I | 0.47 | 0.47 | 0.47 | - | ** | 0.58 |  |
|  | L+G+I | ** | ** | ** | ** | 0.77 | ** | ** |
| *Tribolium castaneum* | G | - |  |  |  |  |  |  |
|  | I | - | - |  |  |  |  |  |
|  | L | 0.47 | 0.47 | 0.47 |  |  |  |  |
|  | L+G | ** | ** | ** | ** |  |  |  |
|  | L+I | 0.24 | 0.24 | 0.24 | 0.58 | ** |  |  |
|  | G+I | 0.24 | 0.24 | 0.24 | 0.62 | ** | 0.94 |  |
|  | L+G+I | ** | ** | ** | ** | 0.54 | ** | ** |
